# Transient knockdown of *Anopheles stephensi* LRIM1 using RNAi increases *Plasmodium falciparum* sporozoite salivary gland infections

**DOI:** 10.1186/s12936-021-03818-8

**Published:** 2021-06-26

**Authors:** Peter F. Billingsley, Kasim I. George, Abraham G. Eappen, Robert A. Harrell, Robert Alford, Tao Li, Sumana Chakravarty, B. Kim Lee Sim, Stephen L. Hoffman, David A. O’Brochta

**Affiliations:** 1 Sanaria Inc, Suite A209, 9800 Medical Center Drive, Rockville, MD 20850 USA; 2 Institute for Bioscience and Biotechnology Research and Department of Entomology, University of Maryland, Gudelsky Drive, Rockville, MD 20850 USA; 3 Present Address: Qiagen Inc, 19300 Germantown Road, Germantown, MD 20874 USA; 4 Insect Transformation Facility, Institute for Bioscience and Biotechnology Research, University of Maryland, 9600 Gudelsky Drive, Rockville, MD 20850 USA; 5 Protein Potential, Suite A209, 9800 Medical Center Drive, Rockville, MD 20850 USA; 6 Foundation for the National Institutes of Health, 11400 Rockville Pike, Suite 600, North Bethesda, MD 20852 USA

**Keywords:** *Anopheles stephensi*, Mosquito, Immune system, Gene silencing, *Plasmodium falciparum*, Oocyst, Sporozoite, PfSPZ vaccine

## Abstract

**Background:**

*Plasmodium falciparum* (Pf) sporozoites (PfSPZ) can be administered as a highly protective vaccine conferring the highest protection seen to date. Sanaria® PfSPZ vaccines are produced using aseptically reared *Anopheles stephensi* mosquitoes. The bionomics of sporogonic development of *P. falciparum* in *A. stephensi* to fully mature salivary gland PfSPZ is thought to be modulated by several components of the mosquito innate immune system. In order to increase salivary gland PfSPZ infections in *A. stephensi* and thereby increase vaccine production efficiency, a gene knock down approach was used to investigate the activity of the immune deficiency (IMD) signaling pathway downstream effector leucine-rich repeat immune molecule 1 (*LRIM1*), an antagonist to *Plasmodium* development.

**Methods:**

Expression of *LRIM1* in *A. stephensi* was reduced following injection of double stranded (ds) RNA into mosquitoes. By combining the Gal4/UAS bipartite system with in vivo expression of short hairpin (sh) RNA coding for *LRIM1* reduced expression of *LRIM1* was targeted in the midgut, fat body, and salivary glands. RT-qPCR was used to demonstrate fold-changes in gene expression in three transgenic crosses and the effects on *P. falciparum* infections determined in mosquitoes showing the greatest reduction in *LRIM1* expression.

**Results:**

*LRIM1* expression could be reduced, but not completely silenced, by expression of *LRIM1* dsRNA. Infections of *P. falciparum* oocysts and PfSPZ were consistently and significantly higher in transgenic mosquitoes than wild type controls, with increases in PfSPZ ranging from 2.5- to tenfold.

**Conclusions:**

*Plasmodium falciparum* infections in *A. stephensi* can be increased following reduced expression of *LRIM1.* These data provide the springboard for more precise knockout of LRIM1 for the eventual incorporation of immune-compromised *A. stephensi* into manufacturing of Sanaria’s PfSPZ products.

**Supplementary Information:**

The online version contains supplementary material available at 10.1186/s12936-021-03818-8.

## Background

Malaria is responsible for over 400,000 deaths a year [1] and despite continued and sustained control efforts, infection rates have plateaued and elimination remains elusive, even in places where *per capita* spending on malaria control is high and advanced programmes are in place [2]. Indeed, the current COVID-19 pandemic is threatening to negate the significant advances that have been made over the last 15 years [3, 4]. New tools are needed in order to progress further towards control and elimination; a vaccine that prevents infection in the human host and thereby transmission to mosquitoes would be the ideal tool. With this specific goal in mind, Sanaria Inc., along with many collaborators, has demonstrated high level protection with two of its *Plasmodium falciparum* (Pf) sporozoite (SPZ)-based vaccines. In clinical trials in 6 countries in Africa, the Germany and the Netherlands in Europe and at 5 sites in the US, PfSPZ-based vaccines have been consistently safe and well tolerated. They have protected > 90% of recipients against controlled human malaria Infection (CHMI) in clinical trials conducted in the USA, Germany, Tanzania, and Mali [5–8] (Sissoko, unpublished) with protection lasting for at least 8 months against heterologous (*P. falciparum* strain 7G8) CHMI [9] and 14 months against homologous (*P. falciparum* strain NF54) CHMI [10]. Protective efficacy of approximately 50% lasting for at least 6 months against naturally transmitted malaria has been demonstrated in four independent clinical trials in Mali [5] (Sissoko and Halimatou unpublished) and Burkina Faso (Sirima, unpublished).

Sanaria Inc. has developed a platform technology for producing aseptic, purified, cryopreserved PfSPZ in compliance with Good Manufacturing Practices [11, 12]. Sanaria® PfSPZ vaccine (radiation-attenuated PfSPZ) [5, 9–11, 13–15], PfSPZ Challenge which is composed of infectious PfSPZ used for CHMI [16–25], PfSPZ-CVac (chemo-attenuated PfSPZ), which combines PfSPZ Challenge with anti-malarial drugs [7, 12, 26, 27], and PfSPZ-GA1 (genetically attenuated PfSPZ) [28] are all reliant on aseptically reared mosquitoes for their manufacture. For all of these products, infection intensity of PfSPZ (number of PfSPZ per mosquito) greatly influences their eventual cost of goods.

One factor controlling PfSPZ infection intensity is the innate immune system of the mosquito. The immune deficiency (IMD) pathway is one arm of the immune system that down-regulates *Plasmodium* infections at the oocyst and SPZ stages, and leucine-rich repeat (LRR) proteins are the downstream effector molecules in the IMD pathway [29]. One LRR, leucine-rich repeat immune molecule 1 (LRIM1), a member of the long LRIM subfamily found only in mosquitoes, is considered a strong suppressor of parasite development playing a role in both melanization and lysis [30–34] of *Plasmodium* ookinetes and oocysts. The current model suggests that LRIM1 functions in a complement-like pathway leading to the activation of a C3-like protein, TEP1, that localizes to the surface of the pathogen, targeting it for destruction [29, 35–37]. LRIM1 covalently binds intracellularly to APL1C forming a heterodimer that is secreted into the hemolymph. The LRIM1/APL1C complex then binds to a mature cleaved TEP1 molecule stabilizing it and promoting binding to the pathogen surface. *LRIM1* expression in *Anopheles gambiae* is regulated by *Plasmodium* infection, with maximum expression coinciding with the movement of *Plasmodium* ookinetes across the midgut epithelium [38–40]. Silencing *LRIM1* expression with dsRNA injected into the mosquito hemocoel increased the intensity of *Plasmodium berghei* oocyst infections 3–4.5 fold in *A. gambiae* [39].

The present study tested the hypothesis that knocking down *A. stephensi LRIM1* would result in higher *Plasmodium* infection intensities at both oocyst and SPZ stages. To achieve this, a transgenic LRIM1 silencer line was produced by crossing a UAS-LRIM1 line to a line expressing the GAL4 transcription activator. LRIM1 expression was reduced but not eliminated and higher infections of *P. falciparum* oocysts and PfSPZ were observed, suggesting that transgenic mosquitoes carrying the knock-down mechanism could be an important approach to increasing the efficiency of manufacture and reducing cost of goods for all PfSPZ products.

## Methods

### Mosquitoes

SDA 500 is a laboratory strain of *Anopheles stephensi* selected for susceptibility to *Plasmodium falciparum* infection [41, 42]. Mosquitoes were maintained in a Conviron environmental chamber at 28 °C, 80% relative humidity and a 12 h:12 h light:dark cycle. Larvae were fed pulverized fish food (TetraMin Tropical Flakes) daily and adults were provided 10% sucrose ad libitum. For colony maintenance, seven-day old adult females were offered a blood meal of bovine blood in acid citrate dextrose (Lampire Biological Laboratories, Pipersville, PA) at 37 °C through a Parafilm membrane using a mosquito feeder (Chemglass Life Sciences, Vineland, NJ). Eggs were collected in 50 mL of deionized water in a 250 mL Biostor multipurpose container (Fisher Scientific, Rockville, MD), lined with Whatman (UK) filter paper.

Artificial feeding buffer composed of 150 mM NaCl; 10 mM NaHCO3; 1 mM Adenosine -5-triphosphate (ATP) [43, 44] was substituted for blood in experiments and fed through a Parafilm™ membrane as described above.

### Infection of *Anopheles stephensi* with *Plasmodium falciparum*

Three separate cohorts of ~ 400 female *A. stephensi* were fed human blood containing stage V *P. falciparum* gametocytes (strain NF54), as described elsewhere [45] and unfed females were removed from the cage. Seven days post infection, midguts ~ 30 mosquitoes from each cohort were dissected and the oocyst intensity determined by microscopy. Fourteen days after blood feeding the salivary glands of ~ 20 mosquitoes were dissected and immediately flash frozen on dry ice for subsequent RNA extraction. The salivary glands of another ~ 30 mosquitoes were dissected, and PfSPZ intensity and prevalence determined.

### Genomic DNA extraction and quantification

Mosquito tissues were homogenized in 50 µL of homogenization buffer (10 mM Tris–HCL pH 7.5, 10 mM EDTA, 5% sucrose [w/v], 0.15 mM spermine, 0.15 mM spermidine) and kept on ice. Fifty microlitres of lysis buffer (300 mM Tris–HCL pH 9.0, 100 mM EDTA, 0.625% SDS [w/v], 5% sucrose [w/v] were added to the homogenized mixture, mixed and incubated at 70 °C for 15 min. The mixture was then cooled to room temperature and 15 µL of 8 M potassium acetate were added, mixed thoroughly then placed on ice for 30 min after which it was centrifuged at 14,000 RPM for 10 min at RT. The supernatant was transferred to a fresh tube and 90 µL of phenol/chloroform/isoamylic alcohol were added. The mixture was centrifuged at 14,000 RPM, 4 °C and supernatant transferred to a new tube and DNA precipitated by adding two volumes of absolute ethanol*.* The mixture was centrifuged at 14,000 RPM for 5 min at RT, supernatant discarded, and the pellet washed in 70% ethanol. After centrifuging for 10 min at 14,000 RPM, the supernatant was discarded and the DNA pellet was vacuum dried then suspended in 1 × TE buffer, pH 7.4. The concentration of nucleic acids was determined using a NanoDrop ND-1000 spectrophotometer (NanoDrop, Wilmington, USA) at 260 nm, and purity checked by measuring the absorption 230 nm and 280 nm.

### RNA extraction and quantification

Total RNA was isolated from mosquito tissues using Ambion Trizol Reagent according to the manufacturer’s instructions. The concentration and purity were determined as above. At 24 h, 48 h and 72 h post feeding, midguts of 20 mosquitoes were dissected into cold phosphate buffered saline (PBS), then midguts and carcasses flash frozen immediately in RNase free tubes on dry ice for subsequent RNA extraction.

### Cloning of *Anopheles stephensi* SDA 500 leucine rich immune molecule 1

LRIM1 was amplified from *A. stephensi* cDNA by PCR using primers AsLRIM1fw (5′-CCC GCC GGT ATA GCT TAT CAG-3′) and AsLRIM1rv (5′-CAA ATA GTG CTC GTC TGC GC-3′). The known *A. gambiae* LRIM1 sequence (AGAP0006348) was aligned using ApE-A Plasmid Editor to an assembled draft genome sequence of *A. stephensi*. Conserved regions between *A. gambiae* LRIM1 and *A. stephensi* LRIM1 [33] were identified and primers designed to amplify the full open reading frame. Phusion High-Fidelity polymerase (New England Biolabs, Ipswich, MA.) was employed for PCR. *LRIM1* PCR product was purified by gel electrophoresis and gel extraction (QIAquick gel extraction kit, QIAGEN, Germantown, MD). Purified PCR product was inserted into Zero Blunt TOPO PCR Cloning vector (Thermo Fisher Scientific, Rockville, MD) according to the manufacturer’s instructions and transformed in *Escherichia coli* DH10B (Thermo Fisher Scientific, Rockville, MD). Positive colonies were digested with EcoRI and agarose gel electrophoresis was used to identify insertion of *LRIM1* PCR product. Sequence identity was then confirmed by DNA sequencing (Macrogen Inc, Rockville, MD).

### Real time reverse transcription PCR

To generate cDNA, 1–5 μg of total RNA were mixed with 1 µL of oligo(dT)20 primer (50 µM), 10 mM dNTP mix and RNase free water to a total volume of 10 μL. The mixture was heated to 65 °C for 5 min and then quickly chilled on ice. A master mix containing 2 μL of 10X reverse transcriptase (RT) buffer; 4 μL of 25 mM MgCl2; 2 μL of 0.1 M DTT; 1 µL of RNase OUT (40 U/µL); 1 µL of Superscript III RT (200 U/µL), was added, gently mixed and incubated at 50 °C for 50 min. The reaction was then inactivated by incubating at 85 °C for 5 min and then chilling on ice. After brief centrifugation, 1 µL of RNase H was added to the mixture and incubated at 37 °C for 20 min. Synthesized cDNA was diluted to 200 ng/µL and used for qPCR. All the samples to be compared were processed in parallel and in triplicate using was an ABI PRISM 7000 Sequence Detection System (Applied Biosystems). Reaction conditions are described in the Additional file 1.

### Synthesis of dsRNA for *LRIM1* silencing

A cDNA fragment of 500 bp of *LRIM1* was amplified using the dsRNAfw and dsRNArv (Additional file 1: Table S3) primers using cDNA from 7-day old *A. stephensi* females as the template. The resulting PCR fragment was cloned into the pCR II-TOPO vector (Invitrogen, Carlsbad, CA) and transformed in *E. coli* DH10B (Thermo Fisher Scientific, Rockville, MD). High yield plasmid DNA was isolated using QIAGEN (Germantown, MD) Plasmid Maxi Kit. The T7 flanked DNA fragment used for dsRNA synthesized was removed from the plasmid by digestion with EcoRI and double stranded RNA was generated and purified using the MEGAscript kit (Ambion, Austin, TX).

### Silencing *Anopheles stephensi LRIM1* by dsRNA injection

Four day old *A. stephensi* females were anesthetized on ice for 5 min and held at 4 ˚C injection plate. Approximately 100 nL of *LRIM1* dsRNA (3 ng/nL) or EGFP dsRNA control were injected into the thorax of the mosquitoes using a Pneumatic PicoPump PV820 (World Precision Instrument Inc., Sarasota, FL). After injection the mosquitoes were allowed to recover at RT for 1 h before being transferred to normal rearing conditions (see above). *LRIM1* silencing was confirmed 4 days post dsRNA injection by qRT- PCR.

For bacterial infections a glass needle was dipped into a pellet of *E. coli* (DH10B) OD600 of 0.1 and injected into the thorax of the mosquito. For feeding experiments, artificial feeding buffer containing *E. coli* at 100 CFU/mL was fed to mosquitoes.

For survival studies, three cohorts of 50 four-day-old adult females were injected as above then held at 28 ˚C, 80% humidity, and 12 h:12 h light:dark cycle with 10% sucrose provided ad libitum. The number of dead mosquitoes were recorded each day. A cohort of 50 untreated mosquitoes served as a second control.

### Vectors

All vectors used in this study are described in Additional file 1.

### Generation of silencer lines

*Anopheles stephensi* preblastoderm embryos were injected with 150 ng/µL vector-containing plasmids and 300 ng/µL plasmids expressing *piggyBac* transposase [46], in 5 mM KCl, 0.1 mM NaPO4, pH 6.8. Insects that hatched and survived to adulthood were pooled according to sex and mated to wild type *A. stephensi* SDA 500. Progeny were screened as larvae for the expression of ECFP or nuclear localization sequence (nls)-EGFP, and transgenic individuals were used to establish lines. The *piggyBac* insertion sites were determined using splinkerette-PCR [47, 48] (Additional file 1: Tables S5, S6). For experiments that required analysis of genetically modified mosquitoes with both the Gal4 transgene and UAS::LRIM1silencer transgene, heterozygous individuals of the UAS::LRIM1silencer and MBL24 GAL4 line were mated to produce progeny with all four genotypes: wild type; MBL24-Gal4/+; UAS::LRIM1silencer/+ and MBL24-Gal4/UAS::LRIM1silencer. MBL24-Gal4/+, UAS::LRIM1silencer/+ and wild type mosquitoes were used as controls.

### Survival comparison of transgenic mosquitoes

LRIM1-silencer/- lines were crossed with the MBL24 Gal4/- driver line. From the progeny, 100 female pupae of each genotype were identified using the fluorescence marker gene. Pupae were pooled, and placed in a 3.8 L mosquito cage. After emergence, the mosquitoes were maintained on a 10 percent sucrose solution. The number of dead mosquitoes were recorded each day.

### Isolation of midgut microbiota for microbial load assessment

Individual *A. stephensi* SDA500 were surface-sterilized by washing three times with alternating 70% ethanol and sterile PBS washes. The midguts were then dissected in PBS using flame-sterilized forceps and homogenized in 200 µL PBS using a sterilized pestle. Each midgut homogenate was then serially diluted and inoculated on Luria–Bertani (LB) agar and incubated at 27 °C for 48 h after which individual colonies counted.

### Statistical analysis

All relative expression data and sporozoite numbers were compared across multiple treatments by ANOVA followed by post-hoc Dunn’s test to identify differences between pairs of treatments. Oocyst data were compared using a Mann Whitney U test followed by Kruskal–Wallis test between pairs of treatments. Survival curves were compared using Mantel–Haenszel chi-squared tests to determine the Odds Ratio. Data were analysed using Graph Pad Prism software V9.1.

## Results

### Cloning of *Anopheles stephensi leucine rich immune molecule 1*

**Fig. 1:**
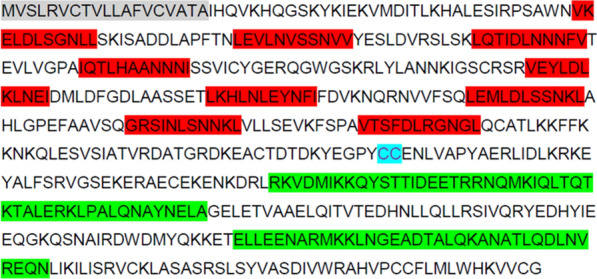
Amino acid sequence and predicted structural organization of *Anopheles stephensi* LRIM1. Grey—signal peptide; Red—leucine rich repeat regions; Blue—cysteine; Green—Coiled coil domains

A 1.8 kb fragment amplified from *A. stephensi* cDNA showed 58% nucleotide sequence identity and 60% amino acid identity to the known *A. gambiae LRIM1*. The sequence contained nine LRR domains consisting of 19–41 amino acid residues, with two coiled coil domains in the region of amino acid residues 318 to 366 and 424 to 459, and a signal peptide region from residue 1 to 19 (Fig. 1). The sequence was identified as the predicted *LRIM1* gene (ASTE000814) when compared to the *A. stephensi* genome (release version VB-2015-10, AsteS1) [49].

### Immune responses of *Anopheles stephensi* to *Plasmodium falciparum infection*

**Fig. 2:**
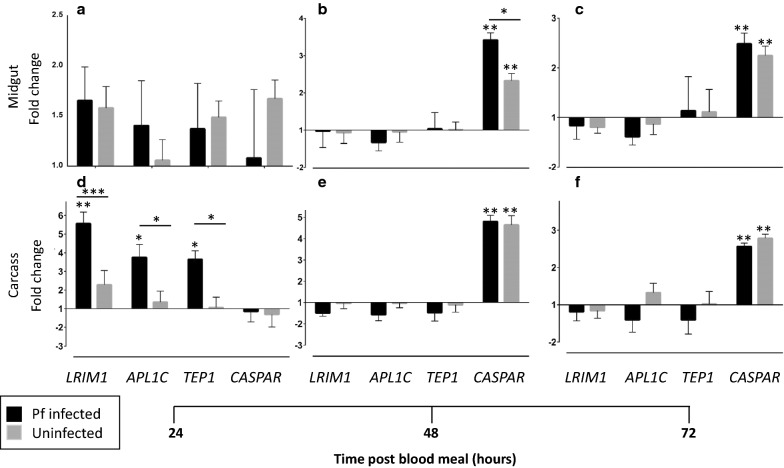
Relative transcript levels of immune genes in adult female *Anopheles stephensi. LRIM1, APL1C, TEP1* and *Caspar* in the midgut (**a**–**c**) and carcass (**d**–**f**) of female *Anopheles stephensi* 24 h (**a**, **d**), 48 h (**b**, **e**) and 72 h (**c**, **f**) after feeding on blood with (black bars) or without (gray bars) *Plasmodium falciparum* gametocytes. Bars represent mean of triplicate experiments ± standard error of mean. *p < 0.05; **p < 0.02; ***p < 0.01. Asterisks above each bar show comparison to baseline; asterisks above horizontal lines show comparison between treatments. Transcript levels are reported as a fold expression compared to naïve non-blood fed females

The midguts and carcasses (i. e. all other tissues) of female *A. stephensi* were assessed at 24, 48 and 72 h post blood meal (hpbm) of human blood alone or human blood containing *P. falciparum* gametocytes, for transcript levels of IMD effector genes *LRIM1*, *APL1C* and *TEP1* and the IMD pathway negative regulator *Caspar*; mosquitoes maintained on 10% sucrose were used as controls against which expression levels were compared. *LRIM1* was upregulated ~ 1.6 fold at 24 hpbm in the midgut independent of infection (Fig. 2a). In the carcass, expression was also upregulated at 24 hpbm, but there was a significant (p < 0.025), almost fivefold increase in *LRIM1* expression associated with infection (Fig. 2d). There was no subsequent significant upregulation or down regulation after feeding in either tissue or in association with infection (Fig. 2b, c, e, f). *TEP1* expression followed the same pattern as *LRIM1* while *APL1C* additionally showed differential upregulation in the midgut associated with infection at 24 hpbm (Fig. 2a). Consistent with its role as a negative regulator of the IMD pathway, *Caspar* transcript levels were elevated in the midgut and the carcass only at 48 and 72 hpbm (Fig. 2).

**Fig. 3:**
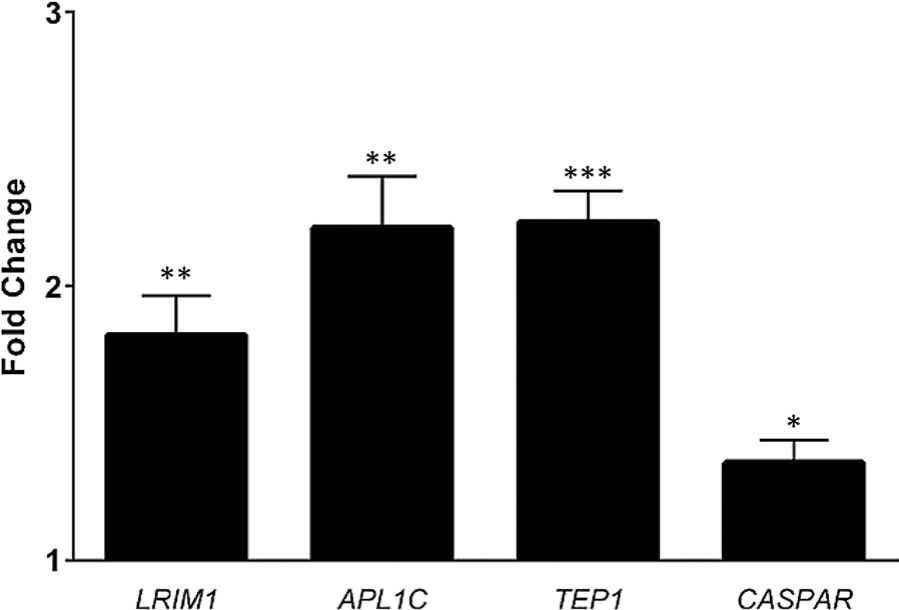
Relative transcript levels of *LRIM1, APL1C, TEP1* and *Caspar* in salivary glands of female *Anopheles stephensi* 14 days post *Plasmodium falciparum* infection. Transcript levels are reported as a fold expression compared to non- infected blood fed females. Error bars indicate standard error of the mean of three independent replicates. *p < 0.05; **p < 0.02; ***p < 0.01. Asterisks above each bar show comparison to baseline

The salivary glands of infected females were assessed for IMD pathway responses fourteen days post *P. falciparum* infection. Both APL1C (p < 0.02) and TEP1 (p < 0.01) showed greater than twofold increase in average transcript levels when compared to mosquitoes that fed on non-infected blood, while LRIM1 showed a 1.8-fold (p < 0.02) increase when compared to controls. There was also a modest, but significant (p < 0.05), 1.3-fold increase in Caspar transcript levels when compared to non-infected blood fed females (Fig. 3).

### Silencing of *A. stephensi LRIM1* by injection of dsRNA

**Fig. 4:**
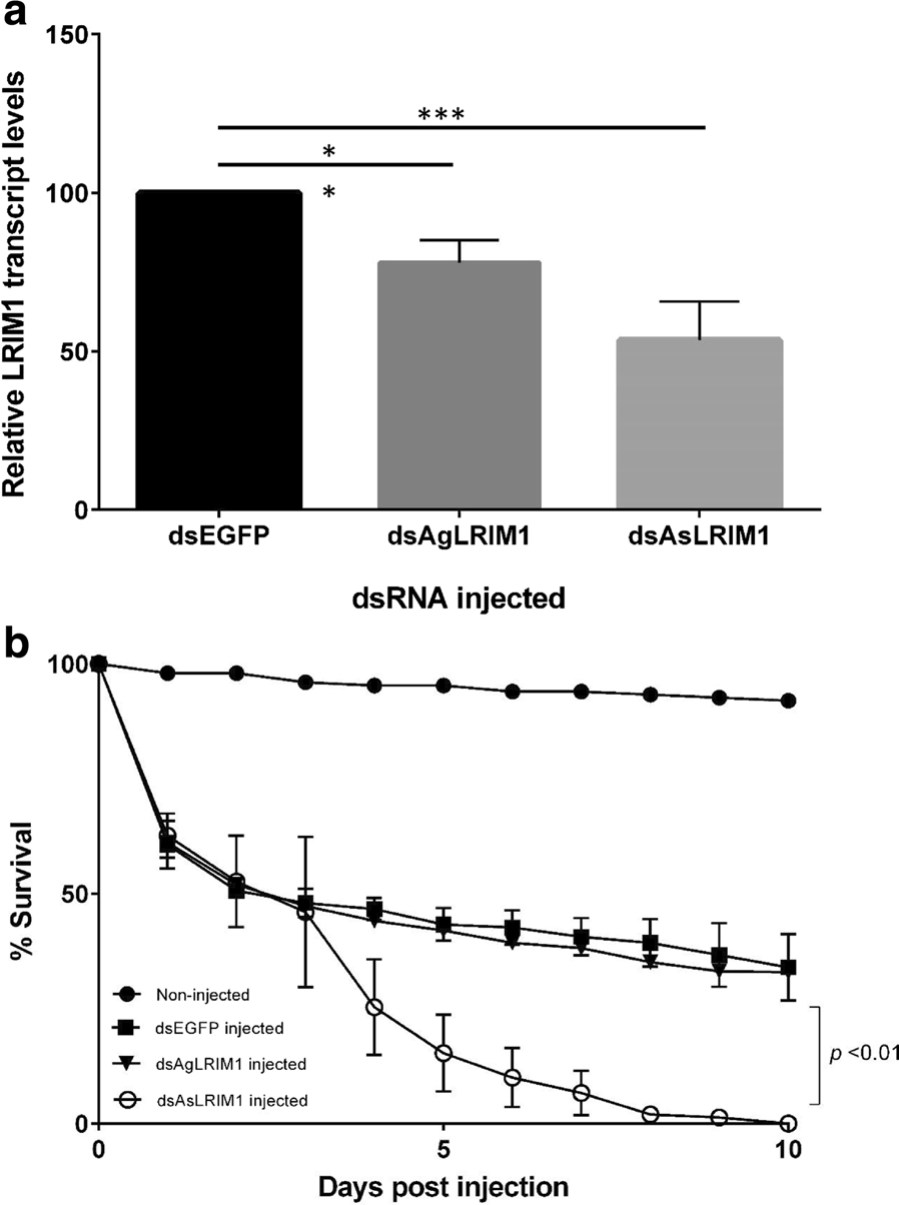
*LRIM1* expression and survival of female *Anopheles stephensi* after dsRNA injection. **a** Expression of LRIM1 in whole mosquitoes 4 days after injection. Average transcript abundance is relative to control mosquitoes injected with dsEGFP. Transcript levels of ribosomal S7 gene were used as a calibrator. Bars indicate standard error of the mean of three independent replicates. **p < 0.02; ***p < 0.01. Asterisks above horizontal lines show comparison between treatments and control. **b** Survival of female *Anopheles stephensi* after injection of dsRNA. Fifty female mosquitoes were used for each treatment. Bars indicate standard error of the mean of three independent experiments

dsRNA injections were used as a preliminary assessment for the silencing of LRIM1 in *A. stephensi*. Female mosquitoes were injected with *dsAgLRIM1* or *dsAsLRIM1* and *LRIM1* expression compared to mosquitoes injected with *dsEGFP*, and silencing of *LRIM1* expression in the whole body was assessed 4 days post injection. *LRIM1* showed reduced transcript abundance of 78.8% and 53.5% after *dsAgLRIM1* and *dsAsLRIM1* injections respectively (Fig. 4a) indicating that *AsLRIM1* could be used as the template for transgenic modification to down-regulate *LRIM1*. However, injecting *A. stephensi* females with dsAsLRIM1 caused 100% mortality in females by 10 days post injection compared to 66 percent and 68 percent fatality of dsEGFP and dsAgLRIM1 injected controls respectively (Fig. 4b).

### Characterization of LRIM1-silencer lines

After confirming the inhibition of *AsLRIM1* following *dsAsLRIM1* injections, the next step was to generate an *LRIM1* silencer line. Three *LRIM1* silencer lines, F2, M2 and M7 were created. The cytogenetic locations of the transgene insertion sites were determined using Splinkerette PCR [48] and the chromosomal location data of *A. stephensi* scaffolds. The integration site for F2 was in the intergenic region of scaffold KB664543 homologous to a locus on chromosome 3R in *A. gambiae*. For M2, the transgene was found in the intergenic region of scaffold KB664524 homologous to a locus on *A. gambiae* chromosome 2R, and the M7 transgene was located in the intergenic region of scaffold KB664832 homologous to a locus on *A. gambiae* chromosome 3L (Table 1).

**Table 1:** Cytogenetic location of the *LRIM1* silencing transgene in the *Anopheles stephensi* genome

Silencer	Insertion Site	
	Scaffold	Chromosome
LRIM1-silencer F2	KB664543	3R
LRIM1-silencer M2	KB664524	2R
LRIM1-silencer M7	KB664832	3L

**Fig. 5:**
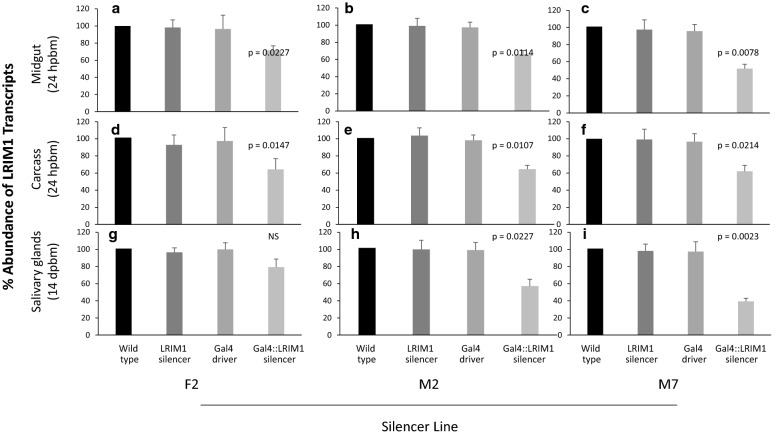
Relative transcript abundance of LRIM1 in driver and silencer LRIM1 mosquito lines. LRIM1 expression in GAL4::LRIM1-silencer lines F2, M2, and M7 was determined in the midguts and carcasses 24 h post blood meal, and salivary glands 14 days post blood meal. For each genotype the midguts or carcasses of 10 females or salivary glands of 30 females were pooled and RNA extracted. Transcript abundance is shown relative to wild type mosquitoes using ribosomal S7 gene as a calibrator. Bars indicate mean and standard error of the mean of three independent experiments

The MBL24 Gal4 driver line expresses Gal4 in the midgut, fat body and salivary gland [50]. The progeny of the UAS:LRIM1-silencer lines were crossed with the driver line and progeny containing a copy of UAS::LRIM1-silencer and Gal4 were used to test for tissue specific silencing of *LRIM1* expression using qRT-PCR. The abundance of *LRIM1* transcript in each genotype was compared to transcript abundance in the wild type. Progeny that contained a single transgene element showed no statistically significant difference in *LRIM1* transcript abundance in tissues examined compared to wild type. Progeny of the three silencer lines (F2, M2 and M7) that contained both the Gal4 and UAS::LRIM1-silencer elements showed reduction of *LRIM1* transcript abundance in the midgut and carcass (midgut and salivary glands removed) (Fig. 5a–f), and M2 and M7 additionally in the salivary glands compared to wild type (Fig. 5h, i). In the midgut, the average transcript abundance was reduced to 72%, 65% and 52% for lines F2, M2 and M7 respectively (Fig. 5a–c). The mean carcass transcript levels in F2, M2 and M7 were reduced to 64%, 65% and 63%, respectively (Fig. 5d–f). For both the M2 and M7 lines, transcript reduction was highest in the salivary glands with average transcript abundance of 56% and 38% respectively (Fig. 5h, i). Based on these results, M7 was down-selected for infection studies.

**Fig. 6:**
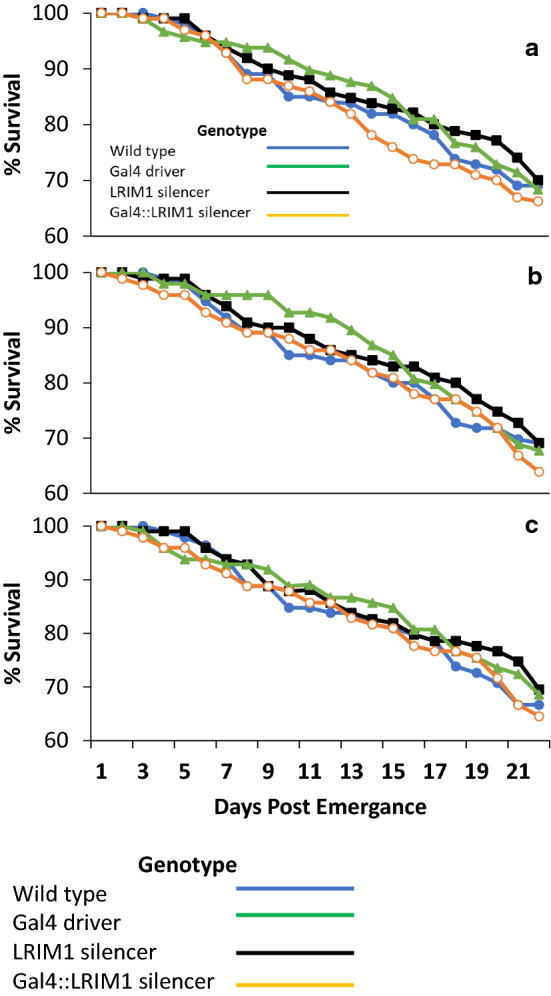
Survival of the progeny from crosses of LRIM1-silencer lines with the MLB24 Gal4 driver line. **a** F2, **b** M2, **c** M7 line. Fifty female pupae from each genotype were pooled and adults observed for 21 days post emergence. The cage was examined daily and dead individuals removed, the genotype determined. No statistical differences were observed among the genotypes examined

Because injection of dsAsLRIM1 into female *A. stephensi* reduced their lifespan, it was necessary to check whether in vivo shRNA silencing with a smaller and more specific target site would have a reduced longevity phenotype. The life spans of the progeny generated from crossing LRIM1- silencer/- lines with the MBL24 Gal4/- driver line were not statistically different in three independent experiments (Fig. 6). In all lines and crosses, 60–70% of female mosquitoes were alive 21 days post emergence.

**Fig. 7:**
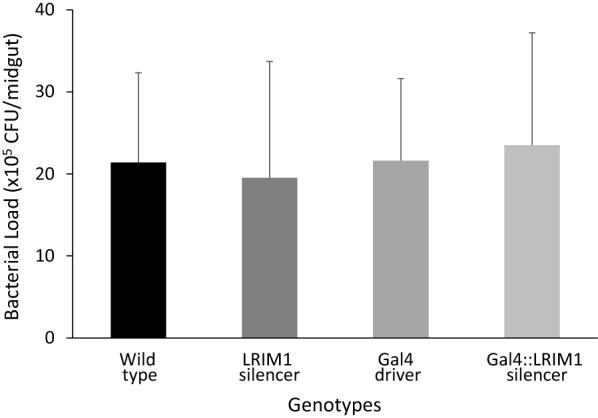
Midgut bacterial load in a cross of LRIM1-silencer M7 with MLB24 Gal4 driver *Anopheles stephensi*. Serial dilutions of midgut homogenate of 10 individual females of each genotype were plated on LB agar. CFUs were calculated after 48 h incubation at 27 °C. Error bars indicate the standard error of the mean of three independent experiments

In addition, and because M7 demonstrated the greatest reduction in midgut expression of *LRIM1*, the bacterial load in the midgut of progeny from the cross of LRIM1 silencer M7 with MLB24-Gal4 driver was determined. No statistical difference was observed in bacterial load of the genotypes examined; the mean number of Colony Forming Units (CFU) in the female midguts ranged from 1.8 × 10^6^ CFU/ml to 2.3 × 10^6^ CFU/mL (Fig. 7).

### *Plasmodium falciparum* infections

**Fig. 8:**
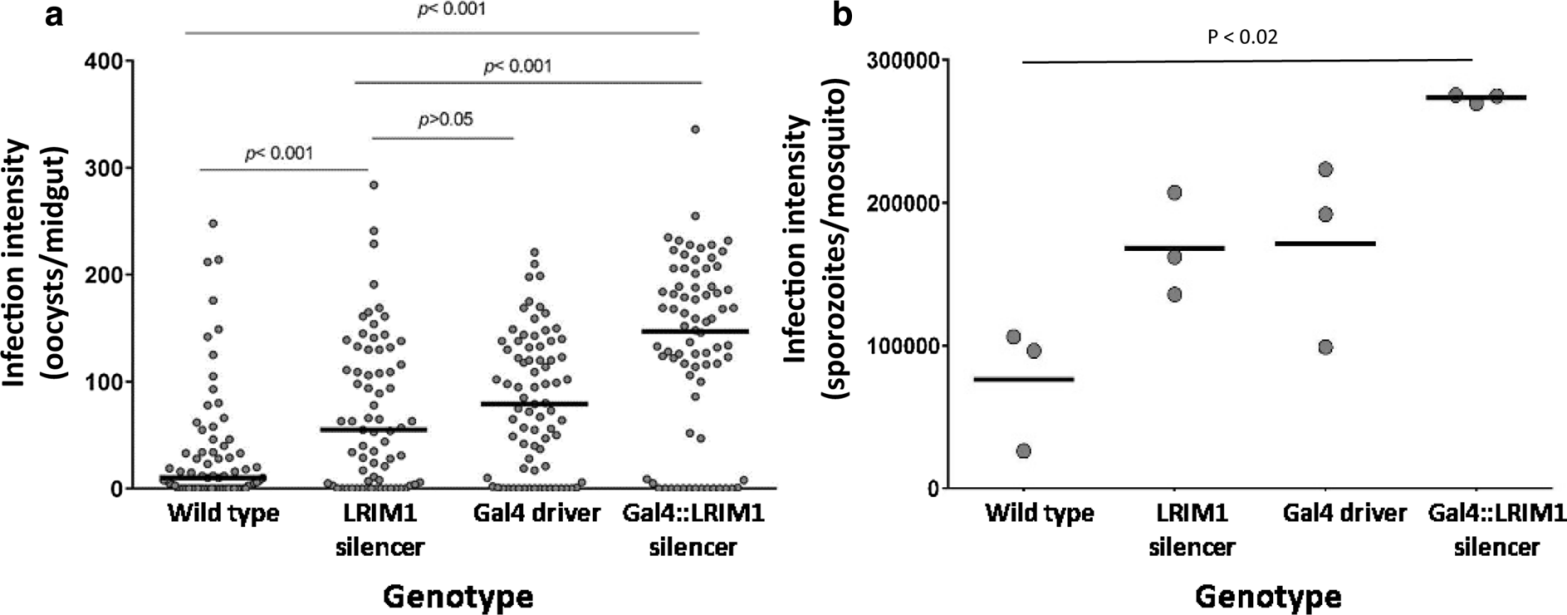
*Plasmodium falciparum* infections in progeny from a cross of LRIM1-silencer M7 with MLB24 Gal4 driver *Anopheles stephensi.* Oocyst infections were determined on day 7 post blood meal and PfSPZ infections on day 21–25 post blood meal. Circles represent the number of oocysts on a single midgut; horizontal black bars represent the median oocysts in each genotype. Three independent biological replicates were pooled, and significance was determined by a Kruskal–Wallis test followed by Dunn’s post-test in the case of multiple comparisons. For PfSPZ, circles represent the mean intensity in a pool of 30 salivary glands from each of three experiments

Heterozygous LRIM1-silencer M7 females were crossed with heterozygous MBL24/Gal4 driver males and the progeny were fed blood containing *P. falciparum* stage V gametocytes. Each resulting genotype was assessed for oocyst and PfSPZ infections. The mean *P. falciparum* oocyst infection intensity of the MBL24/Gal4 UAS::LRIM1-silencer mosquitoes expressing the hairpin silencing construct was 86.0 oocysts/midgut (mean of geometric means for three independent experiments) compared to 8.0 oocysts/midgut in wild type mosquitoes, and 35.7 oocysts/midgut or 35.3 oocysts/midgut in transgenic mosquitoes with only the GAL4 element or UAS LRIM1 silencer element, respectively (Fig. 8a). Mosquitoes expressing the *LRIM1* silencer construct had 2.5–tenfold higher numbers of PfSPZ in the salivary glands compared to wild type. Intermediate PfSPZ intensities were seen in mosquitoes with only the MBL24 Gal4 transgene or the UAS::LRIM1 transgene (Fig. 8b) (Additional file 1: Table S1).

## Discussion

The LRIM1 homolog of *A. stephensi* is a member of the LRIM family, containing the conserved double coiled coil C-terminal domain [30] that is thought to facilitate the protein/protein interactions of LRIM1 and APL1, the resulting heterodimer complex being the effector molecular of the complement-like immune response [35]. Mosquito LRIMs are characterized by a variable number of leucine-rich repeats (LRRs), which distinguishes the short (6–7 LRRs) and long (≥ 10 LRRs) subfamilies of LRIMs. *As*LRIM1 possesses an N-terminal signal peptide indicating that it is a secreted protein; the *Ag*LRIM1 monomer is secreted into the hemolymph only after formation of the LRIM1/APL1 complex. In both *As*LRIM1 and *Ag*LRIM1 between the C-terminal coiled coil domain and the LRRs is a conserved double cysteine motif implicated in the formation of the disulfide bond between LRIM1 and APL1 [30, 36].

*LRIM1* in *A. gambiae* functions as a strong suppressor of *P. berghei* development [32–34], with highest expression observed 24 hpbm, the time at which ookinetes are traversing the mosquito midgut epithelium [39, 40]. *Plasmodium falciparum* infection of *A. stephensi* resulted in a similar transient but significant increase in expression of LRIM1 and other IMD effector molecules at 24 hpbm followed by downregulation at 48 hpbm [39, 40, 51]. The relationship between *A. stephensi* IMD pathway and *Caspar* expression is similar to that seen for *Caspar* and the IMD pathway response to *Plasmodium* in *A. gambiae* [31]. The IMD pathway is clearly induced in *A. stephensi* in response to parasite infections, specifically to *P. falciparum* ookinetes, and functions to limit parasite infections in the mosquito. The midgut immune response, specifically the IMD pathway, during *P. falciparum* infection of *A. gambiae* was infection intensity dependent [31, 52]. The experimental design of the present study did not allow for that relationship to be explored in *A. stephensi*.

The novel observation of a statistically significant increase in expression of IMD effector genes, including LRIM1, in the salivary glands fourteen days post *P. falciparum* infection, when PfSPZ are invading the salivary glands, could provide significant insight into mosquito defense against the parasite late in the sporogonic cycle. Mosquito humoral responses against *Plasmodium* are thought to be concentrated in the midgut, fat body and haemocoel; but these data suggest that, additionally, the salivary glands plus one or more of those tissues in the carcass, can and do mount an immune response against *P. falciparum*. However, it seems most likely that any effects of LRIM1 on PfSPZ may be prior to their full development in the oocyst or after sporulation and oocyst rupture, as parasites are exposed to the hemolymph for several days.

Reverse genetics is an important tool for dissecting aspects of mosquito biology and vector parasite interactions [53, 54]. Transient gene silencing by direct injection of dsRNA and stable expression of hairpin RNAs from transgenes integrated into the genome are two approaches for exploiting gene knockdown or transient silencing using RNAi in mosquitoes [54]. The efficacy of gene silencing by direct injection of dsRNA is severely limited [55–57], being a blunt instrument with which to inhibit tissue- and temporally-specific gene expression. *LRIM1* expression in *A. stephensi* injected with *AsLRIM1* or *AgLRIM1* dsRNA was reduced by46.5% and 21.2%, respectively, demonstrating both the utility and weakness of the approach; while expression was indeed inhibited and the inhibition increased using the species-specific *As*LRIM1, the inhibition was incomplete and short-lived. To address this, the bi-partite Gal4: UAS system was successfully adapted for control of tissue specific in vivo expression of hairpin RNAs in *A. stephensi*. *LRIM1* expression was silenced in the midgut, carcass and salivary glands of *A. stephensi* throughout the entire sexual and sporogonic cycle of *Plasmodium*. However, silencing efficiency was only ~ 40% among the different tissues analysed in three separate *LRIM1* silencing lines. Unlike the blunt instrument of injection, the more refined approach taken here is still imprecise, and optimizing the expression of dsRNA to specific localization, time and quantity of expression of the target gene would require numerous repeats of these experiments based on number and location of the inserted dsRNA. In contrast, the CRIPR/Cas9 gene editing system offers a more surgical, precise method for silencing gene function entirely by disrupting sequence fidelity. Silencing *LRIM1* in *A. stephensi* using CRISPR/Cas9 results in a very different phenotype which will be described elsewhere (Inbar et al., unpublished).

*Anopheles stephensi* injected with *AsLRIM1* dsRNA also had reduced life span [33, 39], a phenotype not observed in mosquitoes expressing in vivo dsRNA *AsLRIM1*. One explanation for this difference is the potential off-target or non-specific effects of the injected dsRNA which represents an overload to the mosquito. Short term high-level inhibition of LRIM1 expression after injection could also allow a transient increase in pathogenic microbiota in the mosquito in response to *LRIM1* silencing, thereby increasing mortality. While the IMD pathway, and specifically TEP1, is considered to play an important role in mosquito defense against bacteria [30, 58–61]; the present results show that silencing *LRIM1* by expression of dsRNA did not change the bacterial load in the midgut. These contrasting observations may explain the differential mortality observed in the two experimental approaches.

*LRIM1* was identified originally as a strong antagonist of *P. berghei*, but not *P. falciparum,* oocysts developing in the midgut of *A. gambiae* [39, 62]. *LRIM1* does in fact contribute to the anti-*P. falciparum* response, but at intermediate oocyst intensities with little effect at low intensities [31]. In the present study, *A. stephensi* expressed a transgene whose transcript formed a shRNA targeting the silencing of *LRIM1*; these mosquitoes had increased intensities of *P. falciparum* and *P. berghei* oocysts as well as PfSPZ and PbSPZ compared to wild type. However, some uncertainty remains concerning the mechanism of increased infections as transgenic mosquitoes containing only the Gal4 transgene or LRIM1 silencer transgene also had increased oocyst and SPZ intensities compared to wild type. It is possible that there was a position effect of the genomic region integrating the MBL24-GAL4 or LRIM1-silencer transgene, though it is unlikely that this would have the same phenotype in both lines. Determination of the insertion sites of the transgenes would provide information concerning the presence, absence or changes to another gene element at or near the insertion site; unfortunately the lines are no longer available for such analyses. Therefore, increases observed in mosquitoes with both transgenes in their genome could be interpreted as an additive effect of the transgenes and not necessarily just *LRIM1* silencing. If increase in PfSPZ intensity is indeed a response to *LRIM1* silencing, then the differences observed between the present data and published studies [31, 39, 62], is due to the approach used for silencing that allowed targeting of *LRIM1* in organs directly involved in the parasite development cycle in the mosquito.

## Conclusions

The survival of all lines of mosquitoes were identical for 21 days post-emergence, suggesting that *LRIM1* knockdown does not affect responses to the main environmental microbial challenges faced by the mosquitoes. Indeed, this is supported by the lack of any effect on both the midgut bacterial population densities. These data, coupled with the more than five-fold average increase in PfSPZ intensities compared to wild type SDA500, suggest that immunocompromised mosquitoes can be developed that could significantly impact Sanaria’s technology platform, increasing manufacturing efficiency and ultimately reducing vaccine cost.

## Supplementary Information

**Additional file 1.** The supplementary materials file contains methods for and results of mosquito infections with *P. berghei*, summary statistics and data for both *P. falciparum* and *P. berghei* infections of mosquitoes, additional molecular methods, and all primers used in the study.

## Data Availability

The data generated and/or analysed during the current study are available from the corresponding author on reasonable request.

## Abbreviations

AgLRIM1: *Anopheles gambiae* LRIM1
APL1C: Anti-*Plasmodium* response leucine rich repeat 1
AsLRIM1: *Anopheles stephensi* LRIM1
ATP: Adenosine 5-triphosphate
cDNA: Complementary DNA
CFU: Colony forming units
CHMI: Controlled human malaria infection
DNA: Deoxyribonucleic acid
ds: Double stranded
dsRNA: Double stranded RNA
ECFP: Enhanced cyan fluorescent protein
EDTA: Ethylenediaminetetraacetic acid
EGFP: Enhanced green fluorescent protein
hpbm: Hours post blood meal
IMD: Immune deficiency
LRIM1: Leucine-rich immune protein 1
PBS: Phosphate buffered saline
PbSPZ: *Plasmodium berghei* sporozoite(s)
PCR: Polymerase chain reaction
RNA: Ribonucleic acid
RPM: Rotations per minute
RT: Room temperature
SDS: Sodium dodecyl sulphate
shRNA: Short hairpin RNA
SPZ: Sporozoite(s)
TE: Tris–EDTA
TEP1: Thioester containing protein 1
Tris–HCL: Trisaminomethane hydrochloride
UAS: Upstream activation sequence(s)
