# Supplementary material for: Transient knockdown of Anopheles stephensi LRIM1 using RNAi increases Plasmodium falciparum sporozoite salivary gland infections
Source: Malar J. 2021 Jun 26;20:284. doi: 10.1186/s12936-021-03818-8 (PMC8235909; doi:10.1186/s12936-021-03818-8)
Supplement: Supplementary file 1 — Additional file 1. The supplementary materials file contains methods for and results of mosquito infections with P. berghei, summary statistics and data for both P. falciparum and P. berghei infections of mosquitoes, additional molecular methods, and all primers used in the study. [file 12936_2021_3818_MOESM1_ESM.docx]

**Supplementary Materials**

**Transient knockdown of *Anopheles stephensi* LRIM1 using RNAi increases *Plasmodium falciparum* sporozoite salivary gland infections**

Peter F. Billingsley, Kasim I. George, Abraham Eappen, Robert Harrell, Robert Alford, Tao Li, Sumana Chakravarty, B. Kim Lee Sim, Stephen L. Hoffman, David A*.* O’Brochta

**Supplementary Results**

***Plasmodium berghei* infections**

Heterozygous LRIM1-silencer M7 females were crossed with heterozygous MBL24/Gal4 driver males and the progeny were fed on *Plasmodium berghei* infected mice. Each genotype was assessed for oocyst prevalence, oocyst intensity and PbSPZ intensity in two separate experiments. Gal4::LRIM1-silencer mosquitoes expressing the hairpin silencing construct had geometric mean oocyst intensities of 60.5 oocysts/mosquito and 17.9 oocysts/mosquito for the two respective experiments (Figure S1). Corresponding intensities for wild type mosquitoes were 41.1 oocysts/mosquito and 12.3 oocysts/mosquito. Transgenic mosquitoes with only the GaL4 transgene or UAS::LRIM1 silencer transgene had mean oocyst counts of 44.2 oocysts/mosquito, and 13.5 oocysts/mosquito and 36.7 oocysts/mosquito and 12.9 oocysts/mosquito respectively. Fourteen days post bloodmeal, mosquitoes were assessed for sporozoite infections (Figure S1). In two independent experiments mosquitoes expressing *LRIM1* silencer construct consistently had increased sporozoite infections compared to wild type with mean sporozoite counts 1.5 to 2-fold higher. Both transgenic controls (Gal4/+ and LRIM1silencer/+) consistently had lower sporozoite counts than Gal4::LRIM1- silencer but not significantly different to wild type control (Table S2).

**Figure S1. Plasmodium berghei infections in progeny from a cross of LRIM1-silencer M7 with MLB24 Gal4 driver *Anopheles stephensi.***  Oocyst infections were determined on day 7 post blood meal and PfSPZ infections on day 21-25 post blood meal. Circles represent the number of oocysts on a single midgut; horizontal black bars represent the median oocysts in each genotype. Three independent biological replicates were pooled, and significance was determined by a Kruskal-Wallis test followed by Dunn’s post-test in the case of multiple comparisons.

**
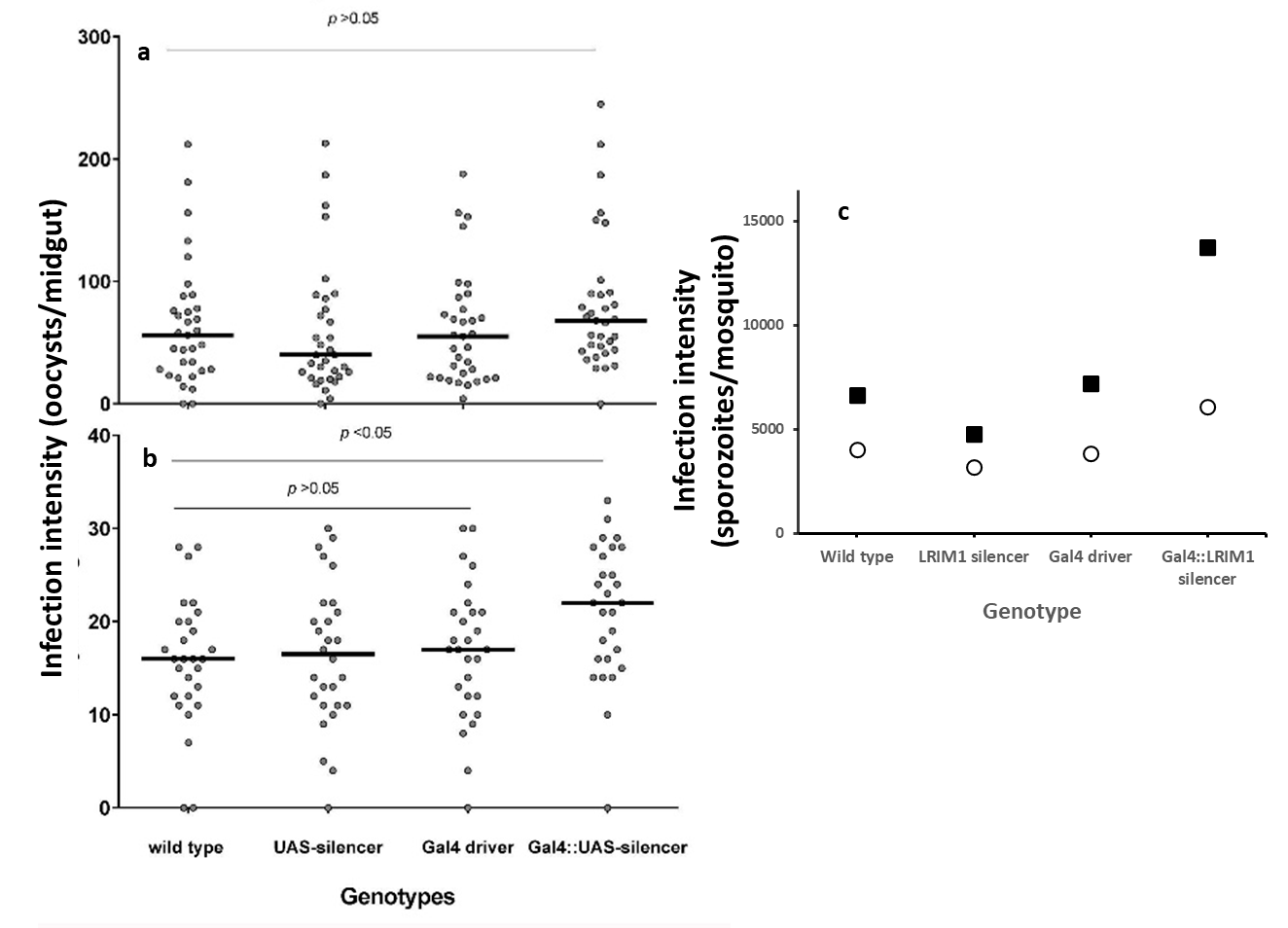
**

**Table S1** **Summary data from *Plasmodium falciparum* infections shown in Figure 8.**

|  | ***P. falciparum* infection** | **Wild Type** | **GAL4 driver** | **GAL4::LRIM1**  **silencer** | **LRIM1 silencer** |
| --- | --- | --- | --- | --- | --- |
| **Feed A** | **Gametocytemia** | 0.5 | 0.5 | 0.5 | 0.5 |
|  | **Prevalence (%) N= 21-25** | 64 | 88 | 79.2 | 77.2 |
|  | **Geomean oocyst/mosquito** | 7.1 | 49.5 | 51.3 | 28 |
|  | **Sporozoites/mosquito** | 26,270 | 191,889 | 274,890 | 207,117 |
|  | **Prevalence (%, head squash; N= 10)** | 44 | 30 | 100 | 56 |
| **Feed B** | **Gametocytemia** | 0.5 | 0.5 | 0.5 | 0.5 |
|  | **Prevalence (%) N= 21-25** | 65 | 70.8 | 96 | 78.3 |
|  | **Geomean oocyst/mosquito** | 9 | 26.7 | 117.8 | 23.8 |
|  | **Sporozoites/mosquito** | 96,365 | 223,483 | 275,438 | 162,060 |
|  | **Prevalence (%, head squash; N= 10)** | 60 | 90 | 100 | 80 |
| **Feed C** | **Gametocytemia** | 0.5 | 0.5 | 0.5 | 0.5 |
|  | **Prevalence (%) N= 21-25** | 62.5 | 84 | 70.8 | 82.6 |
|  | **Geomean oocyst/mosquito** | 8 | 30.8 | 37.5 | 24.5 |
|  | **Sporozoites/mosquito** | 106,210 | 98,980 | 269,733 | 135,800 |
|  | **Prevalence (%, head squash; N= 10)** | 40 | 70 | 100 | 80 |

**Table S2 Summary statistics from *Plasmodium berghei* infections shown in Figure S1**

| ***P. berghei* infection** | **Wild Type** | **GAL4 Driver** | **GAL4::LRIM1**  **Silencer** | **LRIM1 Silencer** |
| --- | --- | --- | --- | --- |
| **Exp.1 Gametocytemia** | 20-24% | 20-24% | 20-24% | 20-24% |
| **Prevalence (%) N= 33** | 93.9 | 100 | 96.9 | 100 |
| **Geomean oocyst/mosquito** | 41.09 | 44.22 | 60.51 | 36.73 |
| **Sporozoites/mosquito** | 6,695 | 7,133 | 13,688 | 4,759 |
| **Exp.2 Gametocytemia** | 20-24% | 20-24% | 20-24% | 20-24% |
| **Prevalence (%) N= 28** | 92.8 | 96.4 | 96.4 | 96.4 |
| **Geomean oocyst/mosquito** | 12.27 | 13.55 | 17.90 | 12.95 |
| **Sporozoites/mosquito** | 4,000 | 3,833 | 6,000 | 3,133 |

**Supplementary Materials and Methods**

**Infection of *Anopheles stephensi* with *Plasmodium berghei***

Mosquitoes were also fed on BAlb/C mice infected with *P. berghei* [1, 2] and each genotype was assessed for oocyst and PbSPZ prevalence and intensity at 7 days and 21 days post feeding respectively. SPZ were released from dissected salivary glands by aspirating in RPMI through a 26 gauge, 2 inch point style 3 Hamilton syringe (Reno Nevada, USA).Ten microliters of SPZ suspension were transferred to a Bright-Line hemocytometer (Hausser Scientific, Horsham Pennsylvania, USA), and after allowing the SPZ to settle for 10-15 minutes, the number of SPZ in two of the four quadrants were counted and the total SPZ calculated.

**Vectors**

**LRIM1-Gal4**

This is a *piggyBac* vector with 672 bases of 5’ terminal and 675 bases of 3’ terminal sequences of *piggyBac* containing the Gal4 ORF under the regulatory control of the LRIM1 promoter in addition to a marker gene encoding enhanced cyan fluorescent protein (ECFP) under the regulatory control of the 3xP3 promoter [3]. This vector was constructed using Gateway recombination cloning technology (Invitrogen, Grand Island, NY), in which 4 recombination modules were simultaneously recombined into a destination plasmid. The first module consisted of the *piggyBac* left terminal with gateway recombination site (Invitrogen, Grand Island, NY). The second module contained Gal4 ORF under control of the LRIM1 promoter, the third module consisted of the ECFP marker gene under the regulatory control of 3xP3 [3] and module four contained the *piggyBac* right terminal. Modules 1, 3 and 4 were present in-house. To make the second module a 3.0 kilobase fragment upstream of the LRIM1 ORF was amplified using primers LRIM1fw 835 (5’- GCG AGG ATG ACC CAC TAG AG-3’) and LRIMrvAscBam (5’-ATA GGA TCC TAG GCG CGC CCC TCC TGA TAA GCT ATA CCG GC-3’) with AscI and BamHI restriction sites and inserted into a PCR4-TOPO vector (Thermo Fisher Scientific Inc., Rockville, MD). A 3.0 kilobase fragment of Gal4hsp3 was amplified from plasmid PB-Gal4 [4] using primers AscI-GAL4fw (5’-ATA GGC GCG CCA GCG CAG CTG AAC AAG CT-3’) and GAL4Rv-BamHI (5’-ATA GGC GCG CCG TAA TAC GAC TCA CTA TAG GGC-3’) and inserted into a PCR Blunt II TOPO vector (Thermo Fisher Scientific Inc., Rockville, MD). The cloning vectors were digested with AscI and BamHI (New England Biolabs (NEB) Ipswich, Mass.) and ligated with T4 DNA ligase (New England Biolabs (NEB) Ipswich, Mass.). Ligated product was transformed into *E. coli* DH10B (Gibco-BRL).

Colonies were screened for insertion. Positive colonies were cultured and plasmid DNA extracted. The LRIM::Gal4 region was amplified from the plasmid using primers attB5-LRIMPromoter835 (5’- GGG GAC AAC TTT GTA TAC AAA AGT TGG GGC GAGGATGACCCACTAGAG-3’) and attB4-SV40Rv (5’- GGGGACAACTTTGTATAGAAAAGTTGGGTGGGTTAAGATACATTGATGAG TTTGGAC-3’that contained with gateway attachment sites. All the modules were brought together during site specific recombination.

**LRIM1-silencer**

This is a *piggyBac* vector with 1.7 kilobases of 5’ terminal and 675 bases of 3’ terminal sequences of *piggBac* containing an inverted repeat of *LRIM1* Gal4 under the regulatory control of the UAS enhancer in addition to a marker gene encoding nuclear localized enhanced green fluorescent protein (nls EGFP) under the regulatory control of the 3xP3 promoter [3]. This vector was constructed using Gateway recombination cloning technology (Invitrogen, Grand Island, NY), in which 4 recombination modules were simultaneously recombined into a destination plasmid. The first module consisted of the *piggyBac* left terminal and a nuclear localized EGFP (Addgene, Cambridge MA, USA) marker gene under the regulatory control of the 3xP3 promoter [3]. The second module contained a 202 base pair region of *LRIM1* juxtaposed to a seventy base pair functional intron and under the regulatory control of the UAS enhancer. The third module contained the inverted repeat of the 200 base pair *LRIM1* region of the second module juxtaposed to SV40. The fourth module contained the *piggyBac* right terminal. Recombination between modules two and three joined the *LRIM1* regions such that transcription resulted in generation of a short hairpin RNA.

To create module one a 300 base pair (bp) region of 3xP3 [3] was amplified using primers NotI-Fse 3xP3fw (5’-GCG GCCGCGGCCGGC CGTTCCCACAATGGTTAATTCG-3’) and PacI-AscI 3xP3rv (5’-GGCGCGCCT TAATTAAGGTACCGTCGACTCTAGC from plasmid attL5-3xP3-EGFP-SV40- attL4. The resulting fragment with NotI/FseI and PacI/AscI restriction sites was inserted into a pCR4 Blunt-TOPO vector (Thermo Fisher Scientific Rockville MD, USA) to create plasmid 3xP3-pCR4. A 1.7 kilobase (kb) region of *piggyBac* left end from an in-house *piggyBac* vector was amplified using primers NotI-PBLeft fw (5’- GCGGCCGCTACATACCTCGCTCTGC-3’) and FseI-PBLeftrv. The resulting amplified fragment with NotI and FseI restriction sites was inserted into a pCR4 Blunt- TOPO vector (Thermo Fisher Scientific Rockville MD, USA) to create plasmid piggBacL-pCR4. A 1.1 kb region of nuclear localized eGFP (nls eGFP) from an in- house plasmid pUAS-Stringer GFP was amplified using primers PacI-nlseGFPfw (5’- TTAATTAAGATCCACCGGTCGCCAC-3’) and AscI-SV40rv (GGCG- CGCCTTAAGATACATTGATGAGTTTGGACAAACC-3’). The resulting PCR product with PacI and AscI restriction sites was inserted into a pCR4 Blunt-TOPO vector (Thermo Fisher Scientific Rockville MD, USA) to generate the nlseGFP-pCR4 plasmid. Restriction digest using NotI (NEB) and FseI (NEB) was performed on both the *piggyBac*L-pCR4 and 3xP3-pCR4 plasmids. The 1.7 kb *piggyBac* left fragment that was generated, was collected and then ligated into a linearized 3xP3-pCR4 blunt plasmid using T4 DNA ligase (NEB) to generate a piggBacL-3xP3-pCR4 plasmid. Restriction digest using PacI (NEB) AscI (NEB) was performed on both the nlseGFP- pCR4 and *piggyBac*L-3xP3-pCR4 plasmids. The 1.1 kb nlseGFP fragment that was generated, was collected and then ligated into a linearized *piggyBac*L-3xP3 pCR4 plasmid using T4 DNA ligase (NEB). The 3.0 kb *piggyBac*L-3xP3-nlseGFP cassette was amplified using primers attB1-PBleftfw (5’-GGGGACAAGTTTGTACAA AAA AGC AGG CTG GTA CAT ACC TCG CTC TGC-3’) and attB5r-SV40 RV (5’- GGG GAC AAC TTTTGT ATA CAA AGTTGT TTAAGATACATTGATGAG TTT GGAC-3’) The amplified fragment with gateway tails was used for a BP reaction with a pDONOR to generate module 1.

Plasmid pSLfa1180 i-CARB-SV40 (Kim, Koo, Richman, Seeley, Vizioli, et al. 2004) was digested with SacI (NEB) and ApaI (NEB) to remove a 1.4 kb region that contained a NotI restriction enzyme site. The overhang ends of the 3.0 kb backbone were blunted using T4 DNA polymerase (NEB) and then re-circularized using T4 DNA ligase (NEB) to form plasmid pSLfa1180 delta. A 202 bp region of *A. stephensi LRIM1* was amplified using primers NheI-NotI-LRIM1fw (5’-GCAGCTAGCGCG GCCGC CGACTGTATC TGGCCAACAATAA-3’) and Xba-LRIM1 RV (5’- CAG TCT AGA GCG GCC GCC TAC GTT CCG CTG GTT CTT-3’) to introduce NheI, NotI and XbaI restriction sites. The amplified fragment was inserted into a pCR4 pCR4 Blunt-TOPO vector (Thermo Fisher Scientific Rockville MD, USA). Plasmid pSL1180 delta and NheI-NotI-LRIM1-XbaI pCR4 were digested with XbaI (NEB) and NheI (NEB). The 200 bp fragment from the digest of NheI-NotI-LRIM1-XbaI pCR4 was inserted into the linearized pSL1180 delta backbone using T4 DNA ligase to make plasmid pSL1180 delta-LRIM1.

A 255 bp region of tdTomato, was amplified and inserted into a pCR4 TOPO Blunt vector. To amplify tdTomato and introduce NheI and NotI restriction sites, primers NheI-tdTfw (5’-GCAGCTAGCGCGGCCGCCGACTGTATCTGGC CA ACAATAA-3’) and NotI tdTrv (5’-ATAGCGGCCGCCTACTTGTAC-3’) were used. The amplified fragment was inserted into a pCR4 Blunt-TOPO vector (Thermo Fisher Scientific Rockville MD, USA). The NheI-tdTomato-NotI pCR4 plasmid and plasmid pSL1180 delta-LRIM1 were both digested with NheI (NEB) and NotI (NEB). The 255 bp fragment of tdTomato released from the NheI-tdTomato-NotI pCR4 plasmid was inserted into a linearized pSL1180 delta-LRIM1 plasmid using T4 DNA ligase (NEB) to form plasmid pSL1180 delta-LRIM1-tdT.

To make module two a 767 bp region of plasmid pSL1180 delta-LRIM1-tdT was amplified using primers attB4-intron-SV40- attB5fw (5’-GGGACAATTTGTAT ACAAAAGTTGCCTAC CACATTTGTAGAGGTTTTACTTGC-3') and attB4_intron_SV40_attB5rv (5’-GGGACAACTTTGTATAGAAAAGTTGGGTGAG GTGAGCACCCAATCATCAG-3'). The amplified fragment with gateway tails was used for a BP reaction with a pDONOR (Gateway, Thermo Fisher Rockville MD, USA) to generate module two.

To generate module three a 457 bp region of plasmid pSL1180 delta-LRIM1- tdT was amplified using primers attB3r-LRIM-tdTomato-attB4rfw (5’- GGGGAC AACTTTATTATACAAAGTTGTCGACTCTGGCCAACA ATAAGAT CG-3’) and attB3r-LRIM_tdTomato-attB4rrv (5’-GGGGACAACTTTTCTATACAAAGTT GGGGGCACGCTGATCTACAAGGTG-3’). The amplified fragment with gateway tails was used for a BP reaction with a pDONOR (Gateway, Thermo Fisher Rockville MD, USA). To generate module 4 a 2210 bp region of an in-house plasmid ECFP-643 was amplified using primers attB3-UAS-PiggBacR-attB2fw (5’-GGGGA CAACTTTGTATAATAAAGTTGCCTATTCAGAGTTCTCTTCTTGTATTC-3’) and attB3-UAS-PiggyBacR-attB2rv (5’- GGGGACCACTTT GTACAAGAAAGCT GGGTAGGTGATGACGGTGAAAACCTC-3’). The amplified fragment with gateway tails was used for a BP reaction with a pDONOR (Gateway, Thermo Fisher Rockville MD, USA). The four modules where then recombined in a LR recombination reaction (Gateway, Thermo Fisher Rockville MD, USA).

**Splinkerette-PCR**

Genomic DNA was extracted from mosquito as described earlier and suspended in 25µL of deionized H2O. 5µL of extracted DNA was digested with BstYI for 2 hours at 60 ˚C in a final reaction volume of 35 µL. Digestion was then heat inactivated at 80 ˚C for 20 mins. 50 µL of SPLINK-BOT and SPLINK-GATC-TOP oligonucleotides were annealed in a NEB Buffer 2 solution of final volume 1000µL by heating at 95 ˚C for 3 minutes then cooled to room temperature. Annealed Spinkerette oligonucleotides are then ligated to digested genomic DNA using T4 DNA Ligase 400U/µL (New England Biolabs (NEB) Ipswich, Mass.) for 2 hours at room temperature. Round one of Splinkerette PCR was carried out using Phusion High-Fidelity polymerase (New England Biolabs (NEB) Ipswich, Mass.) with SPLNK#1 and 3’SPLNK-PB#1 or 5’SPLNK-PB#1 primers.

The PCR reaction was assembled as follows:

Component Volume (µL)

5x HF Buffer 5.0µL

10mM dNTPs 0.5µL

10µM SPLNK#1 primer 0.5µL

10µM 5’ or 3’ SPLINK#1 primer 0.5µL diH20 8.25µL DNA 10µL

Phusion Polymerase 0.25µL

**PCR conditions**

1 cycle: Denaturation 75 s, 98 ˚C

2 cycles Denaturation 20 s, 98 ˚C; Anneal 15 s, 64 ˚C

30 cycles Denaturation 20 s, 98 ˚C; Anneal 15 s, 58 ˚C or 64 ˚C; Elongation 2 min, 72 ˚C

1 cycle Elongation 7 min, 72 ˚C

For the second round of amplification 1µL of the first PCR reaction was carried out using the secondary Splinkerette primers SPLNK#2 and 3’SPLNK-PB#2 or 5’SPLNK- PB#2 under the following conditions:

1 cycle Denaturation 75 s, 98 ˚C

30 cycles Denaturation 20 s, 98 ˚C; Anneal 15 s, 59 ˚C or 66 ˚C; Elongation 90 s, 72 ˚C

1 cycle Elongation 7 min, 72 ˚C

The PCR product obtained was purified by gel electrophoresis on a 1.25% agarose gel then extracted using QUIAGEN, QIAquick gel extraction kit and sequenced using 5'SPLNK-PB-SEQ primer or 3'SPLNK-PB-SEQ primer at Macrogen Inc, Rockville MD.

**Microscopy**

To screen for transgenic mosquitoes by microscopic observation of larvae, pupae, and adults an Olympus MVX10 fluorescent dissecting microscope equipped with Chroma filters (Chroma Technology Corporation, Bellows Falls, VT) 49001 ET- CFP (excitation, 436/20; emission, 480/40; dichroic, 455), 49002 ET-GFP (excitation, 470/40; emission, 525/50; dichroic, 495), 49003 ET-EYFP (excitation, 500/20; emission, 535/30; dichroic, 515), 49005 ET-DsRed (excitation, 545/30; emission, 620/60; dichroic, 570) was used. For tissue imaging a Zeiss Axiom Imager A1 fluorescent compound microscope equipped with Zeiss filter set 20 (excitation, 546/12; emission, 575–640; dichroic, 560) and filter set 38HE (excitation, 470/40; emission, 525/50; dichroic, 495) was used.

**Figure S2. Sequences of LRIM1-from *Anopheles gambiae* and *Anopheles stephensi.***  *A. stephensi* DNA (bottom row) was aligned against the genomic sequence of *A. gambiae* (top row)


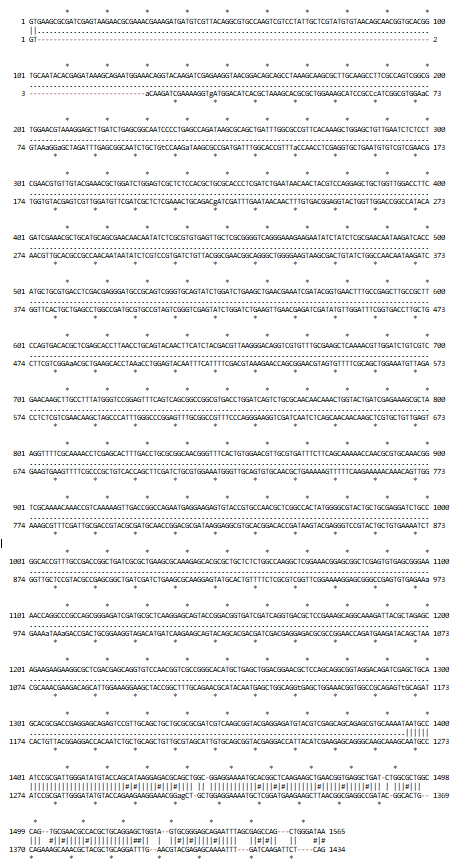


**Table S3. Primers used for quantitative real-time polymerase chain reactions**

| **Name** | **Sequence** |
| --- | --- |
| AsLRIM1-F | GAG GAA AAT GCT CGG ATG AA |
| AsLRIM1-R | CGA CGG CTG AAC CTT ACT GA |
| AsAPL1-F | CTA CAG AGC GAA ATA CAG CA |
| AsAPL1-R | CAG ATG TGC TAT CAC CTT GT |
| AsTEP1-F | TTG CTG TCG TTC GTG ATA |
| AsTEP1-R | AGC GTG ATG GTG TAG TCG |
| AsCaspar-F | TGA CAT CTT CAC CGA AAC GCC |
| AsCaspar-R | AAC TGG ATG CTG CCA ATC GTC T |
| AsRel2-F | GTT CCG CTT CCG CTA TCA GT |
| AsRel2-R | CGC AAC TCT ACC GTG GGG AA |
| tdTomato RT fw | GCG TGA TGA ACT TCG AGG |
| tdTomato RT rv | CCT TGT AGA TCA GCG TGC C |
| GAL1-fw | CCA AAG AAA AAC CGA AGT GC |
| GAL1-Rv | CCC TAG TCA GCG GAG ACC TT |
| AsS7r | TTC GTT GTG AAC CCA AAT AAA AAT C |
| AsS7f | TGC GGC TTC AGA TCC GAG TTC |
| dsRNAfw | CGA CTG TAT CTGGCC AAC AAT AA |
| dsRNArv | AGG AGG CGT GCA CGG ACA CCG A |
| EGFP dsRNA-F | TAA TAC GAC TCA CTA TAG GGA GAG TAA ACG GCC ACA AGT TCA |
| EGFP dsRNA-R | TAA TAC GAC TCA CTA TAG GGA GAC ACG AAC TCC AGC AGG ACC |

**Table S4. Primers used to construct LRIM1-silencer vector**

| Names | sequence |
| --- | --- |
| attB4_intron rv | 5'-  CCCCTGTTGAAACATATCTTTTCAACCCACTCCACTC GTGGGTTAGTAGTC -3' |
| attB5-SV40for | 5’-  GGGGACAACTTTGTATACAAAAGTTGCCATGGTGTA AACATCTCCAAAATGAACG -3' |
| attB3r_LRIM_tdTomato_attB4r fw | CCCCTGTTGAAAAGATATGTTTCAACCCCCGTGCGA  CTAGATGTTCCAC |
| attB3r_LRIM_tdTomato_attB4r rv | GGGGACAACTTTATTATACAAAGTTGTGCTGACATA  GACCGGTTGTTATTCTAGC |
| attB3_UAS_PiggyBacR_attB2 fw | GGGGACAACTTTGTATAATAAAGTTGCCTATTCAGA  GTTCTCTTCTTGTATTC |
| attB3_UAS_PiggyBacR_attB2 rv | CCCCTGGTGAAACATGTTCTTTCGACCCATTTAACCC  TAGAAAGATAATCATATTGTGACG |

**Table S5. Primers used for Splinkerette PCR**

| Names | Sequence |
| --- | --- |
| 5'SPNLK-PB-SEQ | CGA CTG AGA TGT CCT AAA TGC |
| 5'SPLNK-PB#1 | ACC GCA TTG ACA AGC ACG |
| 5'SPLNK-PB#2 | CTC CAA GCG GCG ACT GAG |
| 3'SPLNK-PB-SEQ | ACG CAT GAT TAT CTT TAA C |
| 3'SPLNK-PB#1 | GTT TGT TGA ATT TAT TAT TAG TAT GTA AG |
| 3'SPLNK-PB#2 | CGA TAA AAC ACA TGC GTC |
| SPLNK#1 | CGA AGA GTA ACC GTT GCT AGG AGA GAC C |
| SPLNK#2 | GTG GCT GAA TGA GAC TGG TGT CGA C |
| SPLNK-GATC-TOP | GAT CCC ACT AGT GTC GAC ACC AGT CTC TAA TTT TTT TTT TCA AAA AAA |
| SPLNK-BOT | CGA AGA GTA ACC GTT GCT AGG AGA GAC CGT GGC TGA ATG AGA CTG GTG TCG ACA CTA GTG G |
| SPLNK-Blunt-TOP | CC ACT AGT GTC GAC ACC AGT CTC TAA TTT TTT TTT TCA AAA AAA |

**Table S6. Splinkerette sequence data from transgenic lines**

| **Transgenic Line** | **3' Splinkerette sequence data** |
| --- | --- |
| LRIM1p Gal4M2 | TTAAGTGAGATTTCATGATGACAGTATCTGTTGGCATTAGATTGTAATCGAT  TATTTCAGTTTTCACTCGTAGACTCTCTCCTAATCGAGATC |
| LRIM1p Gal4M4 | TTAGGTCTTCGGAGGTCTCGAGCGAAATGGCAGATGAAGCCCCTCTAATTG  TGTTTGTTAGTAAGACCAACTGGATTCCCCTC |
| LRIM1p Gal4M8 | TTAAGGATGGATTAAGTCAGAGACAAACNGGGAGAAGCAACAAGCAAAAA  AAAAAAAATGCTTCCCCAATT |
|  |  |
|  | **5' Splinkerette sequence data** |
| F2 | TTAACGGTGAGTCGCAACTTCCTGTTGATGCAACCGGGGCGCGCAACATTATCATAGGGTTGCTCCCCTTCCCGAACCGATAGAA |
| M2 | TTAAGGAGCTCAATGAGCAGCAATTTAGGGAAACTTTGCAATCAAAGTGAT GTTTTGTGGTTATCGAAACTACGCAATATGCAAGATA |
| M7 | TTAAATAGCTGCACACAGGCGCATGAGAGATGTGGGTTAAAGGATGGCTGTTCGGGCCAGCGCTATTTATTTGCTACATTTTCAA |
|  |  |
| LRIM1p Gal4M2 | TTAACAACAAAAATGAAAATCGATCCGTATAAAGATC |
| LRIM1p Gal4M4 | NA |
| LRIM1p Gal4M8 | NA |

**References**

1. Dearsly AL, Sinden RE, Self IA. Sexual development in malarial parasites: Gametocyte production, fertility and infectivity to the mosquito vector. Parasitology. 1990;100 Pt 3:359-68.

2. Simonetti AB, Billingsley PF, Winger LA, Sinden RE. Kinetics of expression of two major *Plasmodium berghei* antigens in the mosquito vector, *Anopheles stephensi*. J Eukaryot Microbiol. 1993;40:569-76.

3. Berghammer AJ, Klingler M, Wimmer EA. A universal marker for transgenic insects. Nature. 1999;402:370-1.

4. O'brochta DA, Pilitt KL, Harrell RA, 2nd, Aluvihare C, Alford RT. Gal4-based enhancer-trapping in the malaria mosquito *Anopheles stephensi*. G3 (Bethesda). 2012;2:1305-15.
